# Further Observations on the Case of Miss Margaret M'Avoy

**Published:** 1819-02

**Authors:** Thomas Renwick


*For the London Medical and Physical Journal.*

### *Further Observations on the Case of Miss Margaret M'Avoy;* by Thomas Renwick, M.D.

I DEFERRED addressing you sooner on the subject of Miss M'Avoy's very extraordinary case, from the frequent expectation of its terminating fatally, and of being enabled (if such an event took place) of ascertaining the exact state of disease, and probably the cause of her blindness. Although her present state of health is very precarious, I do not think she is in any very immediate danger, as the abscess has burst four different times, and the matter has passed off with the urine. It is again accumulating; but, as it has found an outlet, it is possible a favourable change in her general health may take place.

Since the 16th of April, Miss M'Avoy has been uniformly ill: afflicted at times with convulsions, hysteria, and loss of speech; constantly complaining of more or less head-ach, and pain in the sockets of the eyes; with very little power of loco-motion. She is scarcely aware of obtaining sleep; or, if she do sleep, the most horrible dreams and night-mare assail her, so as to render the state of waking to be preferable to sleep under these circumstances. She has not lain down in bed since last December, but sits in an arm-chair, or upon a sofa, with her legs sometimes in an horizontal posture, but with her body erect. Her legs and feet swell, and there is a generally leucophlegmatic appearance over her whole body. The catching and oppression in the breathing, and the convulsive motion of the diaphragm, have been so violent as to make it necessary to take from the arm from four to eight ounces of blood at a time; and to repeat the operation upon the recurrence of the symptoms. The bleeding almost uniformly relieved the symptoms, and the blood exhibited a very inflammatory appearance; so that, had this practice not been resorted to, under even discouraging circumstances in the first instance, she must long since have fallen a sacrifice to the violence of the disorder.

Since January last, in forty-one bleedings, she has lost about 245 ounces of blood. The pulse has varied from 108 to 120 pulsations in the minute; but sometimes it has very much exceeded this number.

On the 29th of November, she was seized with convulsions and loss of speech; and again on the 2nd of December; but she did not recover her speech until the 5th. In the evening of the 3d and morning of the 4th of December, she passed from three to four ounces of purulent matter; a part of which is now in my possession.

She has not recovered the power of distinguishing colours, reading, &c. but has sometimes named the colour of metallic substances. I have seen her do this momentarily, in one or two instances; but she lost the power when seized with the catching in the breathing, or upon any sudden spasm attacking her side. She was uncovered. If no fatal event take place previously, it is my intention to publish the continuation of her case early in the spring, with an analysis of the evidence brought against her, and a recapitulation of the more striking proofs in her favour; with a few additional facts which have occurred since the publication of the narrative.

*Liverpool; Dec.* 11, 1818.

P.S. I will thank you to correct the following errors in your Journal for June last:—In the 3d line of page 477, for *addressed* read *adduced*; and in lines 13 and 14 of the same page, for *whenever* read *whatever:* in line 18 of the same page, for *M. Renwick* read *Thomas Renwick.*

